# Single-Cell Sequencing Technology in Ruminant Livestock: Challenges and Opportunities

**DOI:** 10.3390/cimb46060316

**Published:** 2024-05-27

**Authors:** Avery Lyons, Jocelynn Brown, Kimberly M. Davenport

**Affiliations:** Department of Animal Sciences, Washington State University, Pullman, WA 99163, USA

**Keywords:** single-cell RNA-seq, single-cell ATAC-seq, cattle, sheep, goat

## Abstract

Advancements in single-cell sequencing have transformed the genomics field by allowing researchers to delve into the intricate cellular heterogeneity within tissues at greater resolution. While single-cell omics are more widely applied in model organisms and humans, their use in livestock species is just beginning. Studies in cattle, sheep, and goats have already leveraged single-cell and single-nuclei RNA-seq as well as single-cell and single-nuclei ATAC-seq to delineate cellular diversity in tissues, track changes in cell populations and gene expression over developmental stages, and characterize immune cell populations important for disease resistance and resilience. Although challenges exist for the use of this technology in ruminant livestock, such as the precise annotation of unique cell populations and spatial resolution of cells within a tissue, there is vast potential to enhance our understanding of the cellular and molecular mechanisms underpinning traits essential for healthy and productive livestock. This review intends to highlight the insights gained from published single-cell omics studies in cattle, sheep, and goats, particularly those with publicly accessible data. Further, this manuscript will discuss the challenges and opportunities of this technology in ruminant livestock and how it may contribute to enhanced profitability and sustainability of animal agriculture in the future.

## 1. Introduction

Single-cell sequencing technologies have revolutionized the field of genomics, enabling researchers to explore the cellular heterogeneity within tissues at greater resolution [1,2,3]. Among these technologies, single-cell and single-nuclei RNA sequencing (sc/snRNA-seq) and single-cell and single-nuclei assays for transposase-accessible chromatin sequencing (sc/snATAC-seq) have emerged as powerful tools for dissecting the transcriptomic and chromatin landscapes of individual cells [3,4,5]. While these techniques have been more extensively applied across studies in model organisms and humans, their adoption in livestock species is just beginning. Several studies in cattle, sheep, and goats have utilized sc/snRNA-seq and sc/snATAC-seq to characterize cellular heterogeneity within tissues, track cell population and gene expression changes across developmental time, and profile immune cell populations essential for disease resistance and resilience (Table 1, Table 2 and Table 3) [6,7,8,9,10,11,12,13,14,15,16,17,18,19,20,21,22,23,24,25,26,27,28,29,30,31,32,33,34,35,36,37,38,39]. There is immense potential to further utilize these technologies to advance the understanding of cellular and molecular mechanisms governing traits essential for more productive and sustainable livestock while supporting the welfare of these species.

Ruminant livestock including cattle, sheep, and goats play a critical role in global food production; and as the global population is poised to reach 9.7 billion by 2050, the demand for animal products is also increasing [40,41]. Ruminants provide a unique opportunity to convert human-inedible plant material to nutrient-rich foods [40,41]. In order to accomplish the goal of feeding a growing population with fewer animals while using less land and water, ruminant livestock must undergo intense selection aimed at improving traits such as meat quality, milk production, disease resistance, and reproduction [40,41]. Understanding the genetic and biological factors that contribute to these traits at single-cell resolution can provide valuable insights into the genetic and epigenetic factors that drive phenotypic variability [42,43]. Furthermore, the application of single-cell genomics in livestock research has the potential to accelerate the pace of genetic improvement by facilitating the identification of cell-type-specific gene expression, along with gene regulatory elements and networks associated with various traits important to livestock production [6,7,8,9,10,11,12,13,14,15,16,17,18,19,20,21,22,23,24,25,26,27,28,29,30,31,32,33,34,35,36,37,38,39].

Despite the potential benefits, the application of sc/snRNA-seq and sc/snATAC-seq in ruminant livestock species presents challenges. Standardized computational analysis pipelines that are species-agnostic are essential, and the discovery and annotation of cell populations can be difficult in non-model organisms and for tissues such as the rumen, which are unique to these species [44,45,46]. Further limitations of single-cell sequencing include the lack of spatial resolution of cells within a tissue structure and the cost of library preparation [44,45,46]. However, ongoing efforts to improve sequencing protocols, genomic tools and resources, and computational pipelines in livestock are poised to overcome these barriers and pave the way for further use of single-cell sequencing technology in agriculturally important species [47,48,49,50]. This review aims to highlight the findings of published sc/snRNA-seq and sc/snATAC-seq studies in cattle, sheep, and goats that have publicly available data and discuss their impact on the field’s understanding of cellular and molecular mechanisms governing important traits within ruminant livestock. This manuscript will also explore the emerging trends and future directions in the field and how these will contribute to the continued scientific advancement in animal agriculture.

## 2. Brief Overview of Single-Cell and Single-Nuclei mRNA and ATAC Sequencing

**Figure 1:**
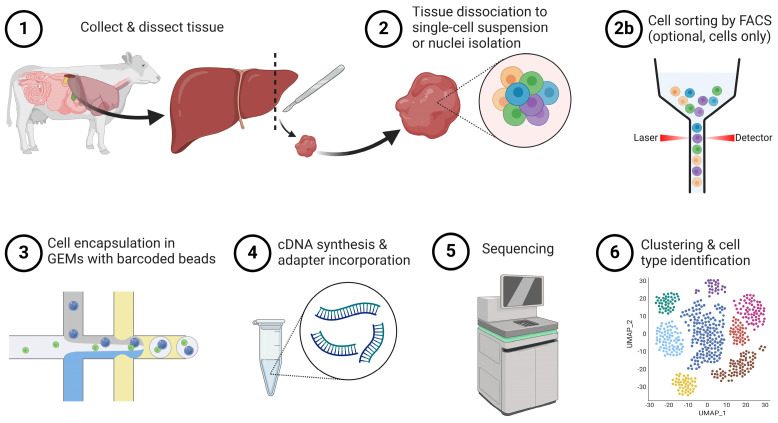
Schematic of single-cell and single-nuclei RNA sequencing workflow (created with BioRender). Abbreviations: FACS (fluorescence-activated cell sorting) and GEMs (gel bead-in emulsions).

Single-cell sequencing, including sc/snRNA-seq and sc/snATAC-seq, is a powerful tool that provides insights into cellular heterogeneity, gene expression profiles across cell types, and regulatory networks. The most commonly used platform for single-cell sequencing in ruminant livestock is currently 10× Genomics Chromium [6,7,8,9,10,11,12,13,14,15,16,17,18,19,20,21,22,23,24,25,26,27,28,29,30,31,32,33,34,35,36,37,38,39]. The 10× Genomics Chromium workflow for sc/snRNA-seq broadly entails cell or nuclei isolation, encapsulation of cells or nuclei in oil droplets with barcoded beads using microfluidics, cell and/or nuclei lysis, and reverse transcription of mRNA while incorporating unique barcodes, adapter ligation, and sequencing (Figure 1) [51]. 

### 2.1. Cell Isolation, Library Preparation, and Sequencing

Cells are first dissociated from whole tissue and can be sorted using fluorescence-activated cell sorting (FACS) to enrich for specific cell types or subset only live cells to be carried forward into sc/snRNA-seq or sc/snATAC-seq library preparation and sequencing [52]. Tissue can also be dissected and snap frozen for nuclei isolation at a later time, which can be advantageous for tissues that can be damaged by enzymatic digestion, are difficult to dissociate into a single-cell suspension such as striated muscle or adipose tissues [53,54,55] or are multinucleated and could be mistakenly filtered out in downstream analyses [19]. Cells or nuclei are then encapsulated in an oil droplet with a uniquely barcoded bead (GEMs; gel in bead emulsions) [51]. For sc/snRNA-seq, cells or nuclei are lysed, and mRNA is reverse-transcribed into complementary DNA (cDNA) while incorporating a unique molecular identifier (UMI) [51]. Sequencing adapters are then incorporated, and libraries are sequenced on either a short- or long-read platform (most commonly short-read) [51]. The sc/snATAC-seq workflow is similar to sc/snRNA-seq and begins with tissue dissociation to single-cell suspension or nuclei isolation from snap-frozen tissue [56,57,58]. Encapsulation with barcoded beads in oil (GEMs) is followed by cell/nuclei lysis and the addition of the Tn5 transposase to fragment-accessible chromatin regions [58]. Sequencing can be performed on many different platforms, but short-read platforms are most commonly used [51]. Multiomic profiling of snRNA-seq and snATAC-seq is also becoming more popular, which provides insight into both the gene expression and chromatin landscape within the same nucleus [59].

### 2.2. Bioinformatic Analyses

There are several tools available for bioinformatic analyses of sc/snRNA-seq and sn/scATAC-seq data. If using a 10× Genomics platform, CellRanger is generally the first step in mapping raw FASTQs to a reference genome and generating count matrices of gene expression across individually barcoded cells [60]. This step will allow the use of any reference genome and is not limited to model organisms. Decontamination of ambient RNA can be incorporated into the bioinformatic workflow and can prove beneficial for snRNA-seq or snATAC-seq datasets, as they can contain a greater amount of ambient RNA carryover from outside the nucleus [60,61,62]. Doublet removal, filtering, and clustering workflows are available through a number of different computational frameworks including Bioconductor [63], Seurat [64], and Scanpy [65] for sc/snRNA-seq and sc/snATAC-seq [60]. After clustering and cell type identification, cell trajectories [66,67,68], RNA velocity [69,70], and cell–cell communication [71,72] can be inferred to gain additional insight into cell population dynamics within the sampled tissue. Additional processing for sc/snATAC-seq datasets can be performed for quality control and doublet removal with specific consideration for sc/snATAC-seq datasets [73,74], followed by peak calling and cell population clustering with packages such as SnapATAC [75], ArchR [76], and Signac [77]. Other informative analyses with sc/snATAC-seq data include identifying differentially accessible regions across cell types, enrichment of transcription factor binding motifs within these regions, and construction of gene regulatory networks [78,79,80]. Within these bioinformatic tools, reference genomes and annotations must be customized to livestock genomes. The tools listed here allow for the creation of custom genomes and annotation, but whether a tool or pipeline allows for custom genomes and annotation is a major consideration for livestock researchers.

### 2.3. Data Interpretation

Performing sc/snRNA-seq and sc/snATAC-seq results in large datasets that can define cellular heterogeneity within complex tissues, model cell differentiation and RNA velocity across developmental time, identify differentially expressed genes and differentially accessible chromatin regions across cell populations, and many other applications. Acquiring both RNA and ATAC datasets provides an opportunity to integrate and putatively link gene expression changes to differentially accessible chromatin regions, likely housing regulatory elements, and construct gene regulatory networks with transcription factor binding sites [78,79,80]. However, open chromatin data only define the accessibility of putative regulatory regions and do not specify which type of regulatory element (i.e., promoter or enhancer) resides within that region (histone modification data will indicate. Further, putative regulatory elements for specific genes can only be identified in a linear context and interpreted in *cis*, as there is no three-dimensional genome information provided by these datasets.

In summary, the library preparation and bioinformatic analysis workflows for sc/snRNA-seq and sc/snATAC-seq include tissue dissociation or nuclei isolation, individual barcoding of cells or nuclei, sequencing, unsupervised clustering of cells and population identification, and further bioinformatic analyses to elucidate trajectories and regulatory networks. There are many different platforms and tools to accomplish library preparation and data analysis goals, and standardizing quality control metrics and data processing will become even more important as this technology is more widely adopted in livestock research. Data from these technologies provide insight into cellular heterogeneity and differentially expressed genes across cell populations of complex tissues among other applications; however, they have limitations in precisely identifying the type of regulatory element associated with gene expression changes.

## 3. Current Uses of Single-Cell Sequencing in Ruminant Livestock Research

**Figure 2:**
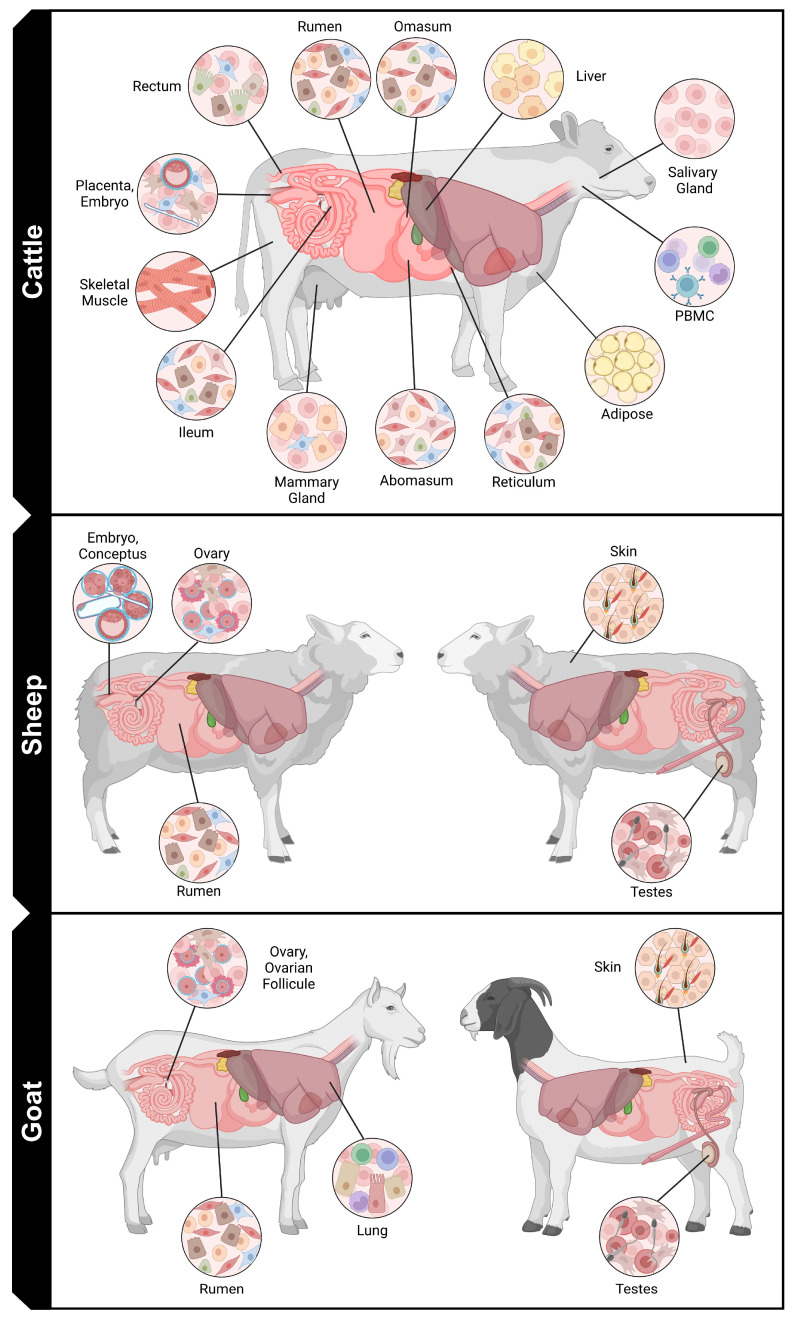
Schematic of tissues sampled for single-cell gene expression and chromatin accessibility assays in cattle, sheep, and goats in published studies with publicly available datasets (created with BioRender). Abbreviations: PBMC (peripheral blood mononuclear cells).

As with studies in model organisms and humans, single-cell sequencing can reveal the underlying heterogeneity of tissues or cells and identify cell-specific regulatory elements and biomarkers in ruminant livestock tissues (Table 1, Table 2 and Table 3, Figure 2). It can also track cell populations and gene expression across developmental time and provide precise insights into the expression and chromatin accessibility of immune cell populations essential for resistance and resilience to disease. Research in ruminant livestock has made significant advancements to date, and scientists will likely continue to employ this technology to discover novel associations with economically important traits.

### 3.1. Cellular Heterogeneity within Tissues Important for Food and Fiber Production

The enhanced resolution provided by single-cell sequencing has been instrumental in identifying rare cell populations, such as stem cells, immune cells, and specialized tissue-specific cells, which play critical roles in development, growth, and disease resistance in livestock. One study identified 55 cell types across the abomasum, ileum, liver, mammary gland, omasum, rectum, reticulum, rumen, salivary gland, and isolated PBMCs in cattle [6]. All of these tissues in ruminant livestock play a critical role in converting human-inedible plant material into nutrient-rich milk and meat products [6]. This study constructed a single-cell atlas of these tissues in dairy cattle, which provided insight into the cellular heterogeneity and complexity within each tissue, and identified key cell types including epithelial and immune cells involved in milk production [6]. Because milk production is a critical aspect of the dairy industry, another study in dairy cattle performed scRNA-seq of cells isolated from fresh milk at mid-lactation to characterize cell types and gene expression [13]. This study identified many different cell types isolated from milk, including immune cells (T-cells, neutrophils, B-cells, monocytes, macrophages, and others) as well as luminal epithelial and progenitor cells [13]. An additional study performed scRNA-seq from a similar isolation of somatic cells from milk as well as primary bovine mammary epithelial cells in culture [20]. Both immune and epithelial cell types were identified from scRNA-seq from milk, while cell populations from the original tissue source (ductal region of the udder) were identified from cultured primary mammary epithelial cells [20]. These studies lay the groundwork for further examining cell types critical for lactation in dairy cattle.

In addition, transcriptomically distinct cell populations of the rumen, a tissue that is critical for digestion and feed efficiency and is unique to ruminants, were identified using scRNA-seq in sheep [27,28] and goats [28] as well as cultured primary epithelial cells in cattle [21]. In cattle, six cell populations were identified from cultured primary ruminal epithelial cells [21]. Cell lineages were then modeled across developmental time, and regulatory networks were constructed to better understand the unique gene expression profiles of these cells and their roles in rumen development in function [21]. A study in sheep examined changes in rumen cell populations and gene expression before, during, and after the development of papillae by sampling lambs during prenatal and early postnatal development [27]. Cell populations identified from this study included basal cells and keratinocytes, and marker genes for papillary growth and development in the rumen were characterized [27]. These results provide critical information about cellular dynamics during the establishment of rumen papillae, which are critical for digestion in sheep and other ruminants [27]. A comparative study of rumen maturation in sheep and goats identified cell populations at different developmental time points [28]. This study characterized cellular communication and transcription factor networks critical for rumen development, and correlated changes in cell populations across development to changes in the rumen microbiome [28]. Overall, studies examining cell populations in the rumen across development and maturation, including potential interactions with the microbial populations, provide valuable insights into biological processes essential for rumen development and ultimately the capacity to convert human inedible feedstuffs into nutrient-rich meat and milk products for consumption.

Other agriculturally important traits in ruminants such as growth and fat deposition were investigated in cattle by performing scRNA-seq and scATAC-seq in skeletal muscle [14,15], scRNA-seq of cultured bovine satellite cells [22,23], and snRNA-seq of adipose tissue [7], which uncovered previously unrealized cellular heterogeneity. Both scRNA-seq and scATAC-seq were employed in the skeletal muscle of cattle at fetal, postnatal, and adult stages of development [15]. This study identified several transcriptionally distinct cell types within skeletal muscle along with putative regulatory elements in open chromatin regions, cell–cell communication pathways, and transcription factor regulatory networks [15]. Another study used scRNA-seq in skeletal muscles of Wagyu, Brahman, and Wagyu/Brahman crosses to examine the cell types potentially involved with the deposition of intramuscular fat and connective tissue [14]. By using these specific breeds, the study was able to identify unique cell types predicted to be involved with enhanced intramuscular fat deposition (Wagyu) and intramuscular connective tissue deposition (Brahman), which both influence meat quality [15]. Further, single-cell RNA-seq of bovine satellite cells in culture isolated from a 2-week-old calf revealed different cell populations expressing markers of adipose progenitor cells that contribute to intramuscular fat deposition [22]. Another study recapitulated these findings from bovine satellite cells in culture and further characterized fibro-adipogenic precursor cells using scRNA-seq [23]. These studies are critical first steps in understanding underlying cellular heterogeneity in skeletal muscle and how different cell types contribute to desirable traits such as increased growth or greater meat quality in ruminants.

Wool and hair quality are traits important to sheep and goat production across the world. The skin and hair follicle cell populations were characterized in sheep [29] as well as cashmere goats [36,37,38]. In sheep, differences in wool curvature due to hair follicle cell types were compared using scRNA-seq [29]. This study revealed differences in gene expression signatures of dermal papilla cells between curly and non-curly wool that may regulate curvature [29]. Several studies in cashmere goats identified heterogeneity of dermal papillae from different follicle types during fetal development [36], between goats producing course and fine cashmere [37], and during different times of the year corresponding to specific hair growth periods [38]. These studies provided insight into hair follicle development across different developmental time points, hair characteristics, and stages of the growth cycle, which are all important for the production of quality fiber in sheep and goats as well as other ruminants.

### 3.2. Gene Expression Dynamics during Reproduction and Development at Single-Cell Resolution

Reproductive efficiency in livestock is essential for profitable and sustainable farms. Previous work characterized the development of gonads at the fetal stage in cattle [12] and goats [32] with scRNA-seq and scATAC-seq, respectively, and compared these cell populations with other mammalian species. Cell populations of mature female gonads including the ovary in sheep [26] and goats [35] as well as the ovarian follicle in goats [34] were also identified with scRNA-seq. Heterogeneity of testes was identified with scRNA-seq in sheep [30,31] and goats [39]. Overall, these studies provide insights into gonadal and germ cell development in ruminants, which facilitate successful reproduction.

Single-cell sequencing has also proven useful for detangling dynamic gene expression within specific cell populations during embryonic and placental development in ruminants. Cellular heterogeneity was uncovered during early embryonic development at the 8- and 16-cell stage in cattle, which coincides with embryonic genome activation [8]. Cell populations and gene expression at single-cell resolution were also investigated during embryonic and conceptus development in sheep, providing additional information on biological processes contributing to pregnancy establishment in ruminants [24,25]. In vitro, scRNA-seq profiled gene expression across cell types in bovine trophectoderm outgrowth of embryos in culture [11] and bovine embryos that had undergone somatic cell nuclear transfer [10]. Further, cell population and gene expression dynamics of bovine embryos pre- and post-implantation, as well as the developing and mature placenta, provided insight into important processes governing implantation and placentation in cattle, which is critical for successful pregnancy establishment and maintenance [9,18,19]. Successful pregnancy establishment and maintenance are critical for the sustainability of ruminant livestock production.

### 3.3. Immune Cell Gene Expression and Chromatin Profiling

Disease resistance and resilience are very important for healthy animals and sustainable livestock production. scRNA-seq and scATAC-seq were performed on peripheral blood mononuclear cells (PBMC) in cattle, including cellular responses to lipopolysaccharide (LPS) challenge [16,17]. Further, snRNA-seq was utilized with lung tissue from 11 non-model species including goats, which revealed conserved cell populations and gene expression across mammals [33]. Future studies using single-cell sequencing technologies in immune cells and tissues, including their responses to disease challenges, have vast potential for identifying molecular mechanisms governing disease resistance and resilience in ruminant livestock species.

The field of single-cell genomics is rapidly evolving, and new datasets are continually being generated. Public data repositories such as the National Center for Biotechnology Information (NCBI), the Gene Expression Omnibus (GEO), the European Nucleotide Archive (ENA), and the Functional Annotation of Animal Genomes (FAANG) Data Portal are all currently being used to deposit raw data. A list of publicly available sc/snRNA-seq and sc/snATAC-seq datasets for cattle (Table 1), sheep (Table 2), and goats (Table 3) is provided. As these technologies continue to evolve and become more accessible, they hold tremendous promise for transforming our understanding of genetics, breeding, and disease resistance in ruminant livestock species and beyond. Ultimately, this will contribute to sustainable and efficient livestock production systems.

## 4. Challenges and Limitations of Single-Cell Technologies in Ruminant Livestock Research

Despite the transformative impact of sc/snRNA-seq and sc/snATAC-seq on livestock genomics research, these technologies are not without limitations. Understanding these challenges is crucial for the accurate interpretation and application of single-cell data in biological contexts. Technical variability, bioinformatic challenges in non-model species, cost, spatial resolution of cell populations, and consistency of data and metadata submitted to public repositories all pose challenges. However, initiatives within the genetics and bioinformatics community are poised to address many of these challenges.

### 4.1. Technical and Bioinformatic Challenges

One of the technical challenges associated with sc/snRNA-seq and sc/snATAC-seq is the presence of technical variability and dropout events during library preparation and sequencing, which can lead to the incomplete detection of gene expression or accessible chromatin regions [44,81,82]. Dropout events occur when the expression level of a gene or accessibility of a region falls below the detection threshold, leading to false negatives and potential biases in downstream analyses [81]. Livestock species often exhibit high levels of genetic diversity and complex transcriptomic and chromatin profiles, which can exacerbate the impact of technical variability and dropout events, making it difficult to accurately quantify gene expression levels and identify differentially expressed genes [45]. This could influence the reliability and reproducibility of single-cell sequencing data, providing further challenges in interpreting downstream results and comparing findings across studies. As library preparation, sequencing, and analysis algorithms continue to improve, these challenges will likely be mitigated.

Many different tools and pipelines exist for the analysis of sc/snRNA-seq and sc/snATAC-seq datasets, with more being developed and released regularly. This speaks to the rapid advancement of the field; however, there is a lack of standardized pipelines for quality control, filtering, and clustering, especially for non-model organisms. This can prove challenging for comparing and reproducing results across studies [60,83]. Using a variety of computational languages to analyze data can pose challenges for researchers if this is not their area of expertise. The analysis of sc/snRNA-seq and sc/snATAC-seq data also presents bioinformatics challenges in non-model species, including ruminant livestock. Many analysis tools built for model organisms are expanding to become species-agnostic, which provides substantial opportunities for livestock researchers to utilize these with annotation from their own genomes. The quality and utility of ruminant livestock genomes and annotation are advancing rapidly driven by community efforts, including pangenome construction [47,48,49,50], complete telomere-to-telomere assemblies [84], and sophisticated annotation pipelines and resources [85]. These efforts will enhance the ability to precisely differentiate cell clusters based on gene expression, providing even greater insights into cellular heterogeneity and gene expression patterns within tissues important for the understanding of economically important traits in ruminant livestock.

### 4.2. Cell Cluster Identification and Spatial Resolution

Cell cluster identification and naming can be a challenging aspect of single-cell data analyses. This step in the analysis is critical, as it leads to many novel discoveries; however, it can prove to be the most difficult in non-model species, especially in tissues such as the placenta, which are highly specialized structurally across mammals [18,19]. Currently, cell populations in non-model organisms such as livestock are frequently identified with the top differentially expressed genes for each cluster, using databases such as the Human Protein Atlas [86] and PanglaoDB [87], or automated cluster identification using databases built from humans and mouse cell clusters [88,89]. Often, cell types are classified as “unknown” if no definitive answer can be reached. One potential new way to combat these obstacles is to use generative pre-trained transformers (GPT) to identify single cell clusters, which could shift the annotation process from manual to semi- or fully automated [90]. Using GPT was deemed to be competent in naming clusters and identifying marker genes with a 70% match rate in most tissues in a recent study [90]. As artificial intelligence technology continues to evolve, it could be incorporated into single-cell omics pipelines to identify cell clusters.

Another limitation of sc/snRNA-seq and sc/snATAC-seq is the lack of spatial resolution, which impedes the precise characterization of cell–cell interactions in spatial context and organization within tissues and organs [91]. Cell populations identified from single-cell sequencing have been localized in a spatial context using techniques such as RNA in-situ hybridization and immunofluorescence [19,92]. In livestock species, where tissue architecture and cellular microenvironments play crucial roles in development, growth, and disease resistance, the inability to capture spatial information can limit the biological insights gained from single-cell sequencing data. Emerging spatial transcriptomics and spatial genomics technologies offer promising solutions to this challenge by enabling the profiling of gene expression within intact tissue sections [91]. These technologies are rapidly improving in robustness and resolution and are becoming more widely utilized in non-model organisms [93,94]. Spatial omics technologies have vast potential for ruminant livestock research by providing spatial resolution and insights into cell–cell interaction and communications in tissues important for production such as the rumen.

### 4.3. Reproducibility, Collaboration, and Knowledge Sharing

The availability and re-use of single-cell genomics datasets are important for answering important biological questions in ruminant livestock research. The accessibility of high-quality, well-annotated datasets accelerates new discoveries and enhances the reproducibility and robustness of the initial single-cell atlases [83,95,96]. Data availability and re-use enable researchers to build upon existing knowledge and leverage the collective insights gained from multiple studies, thereby accelerating scientific discoveries and facilitating the identification of novel biomarkers, regulatory elements, and gene networks associated with economically important traits. By accessing and utilizing existing datasets, researchers can validate and extend previous findings, generate new hypotheses, and explore biological questions more efficiently and cost-effectively [47,83,95]. Further, the availability of single-cell omics datasets across different species, breeds, and experimental conditions facilitates comparative analyses, enabling researchers to identify conserved and species-specific gene expression profiles and regulatory networks associated with complex traits and biological processes in livestock and across species [97,98,99].

In summary, single-cell sequencing technologies can provide valuable insights into important biological questions in livestock species; however, these technologies are not without challenges or limitations. Single-cell library preparation and sequencing can be quite costly and more resource-intensive than traditional bulk sequencing methods; however, the increase in throughput and the decrease in cost per sample for library preparation and sequencing as this technology evolves will enable greater adoption and accessibility of single-cell sequencing in ruminant livestock research [47,100]. Continued collaborative efforts to develop better tools and standardized pipelines for sample preparation, analyses, and data submissions to public databases are essential for realizing the full potential of single-cell omics technologies in ruminant livestock [48,96,101].

## 5. The Future of Single-Cell Sequencing in Ruminant Livestock Research

Despite the current limitations, the rapid advancements in single-cell genomic technologies offer promising opportunities for research in ruminant livestock genetics and physiology, encompassing economically important phenotypes spanning from reproduction and growth to disease resistance. This in turn has tremendous potential for addressing key challenges and driving innovation in the agricultural sector, contributing to sustainable and profitable animal agriculture globally.

Continued development of single-cell gene expression atlases across complex, heterogenous tissues and cell populations in livestock across developmental time will provide greater insights into cell-type-specific expression dynamics. One very promising use of single-cell omics sequencing in livestock research is the comprehensive profiling of immune cell populations and their interactions within the host [16,17,102]. Understanding the dynamics of immune cell heterogeneity and function is crucial for developing strategies to improve disease resistance in livestock. Single-cell omics enables precise characterization of immune cell subsets, including T cells, B cells, macrophages, and dendritic cells, and the identification of cell-type-specific gene expression signatures and regulatory networks associated with immune response and pathogen resistance [16,17,102]. Future research leveraging single-cell omics could focus on elucidating molecular mechanisms underlying host–pathogen interactions, vaccine efficacy, and immune system development in livestock. Another promising application of single-cell technologies is further examining the interaction between host and microbes in terms of the microbiome by expanding on previous work associating cell populations of the rumen with the microbial population composition in sheep and goats [28].

The development of single-cell isoform sequencing and epigenetic assays, along with spatial transcriptomics, also hold promising applications in ruminant livestock research. Single-cell isoform sequencing enables precise identification of alternate isoform expression across cell types within a tissue [103]. Single-cell implementations of CUT&TAG [104], chromatin structure [105,106], and methylation [107,108] also provide opportunities to improve the functional annotation of different cell types within complex tissues [47] when integrated with sc/snRNA-seq and sc/snATAC-seq. Spatial transcriptomics also offers exciting opportunities to localize the spatial context of gene expression and better characterize cell–cell communication [93,94]. Future studies could leverage multiple single-cell omics sequencing technologies to elucidate regulatory networks, contributing to complex traits such as growth, milk production, meat quality, and reproduction in livestock. This will facilitate the development of more precise breeding strategies for livestock operations to increase the productivity and sustainability of animal agriculture.

## 6. Conclusions

Ultimately, incorporating single-cell omics technologies into livestock research holds the potential to facilitate new discoveries to accelerate the pace of genetic improvement, enhance disease resistance, support the welfare of these species, and improve the overall profitability and sustainability of livestock production. Ruminant livestock researchers have already implemented sc/snRNA-seq and sc/snATAC-seq across multiple tissues to uncover cellular heterogeneity, track gene expression dynamics across developmental time, and profile immune cells. Although several limitations to this technology exist, including the lack of spatial resolution, cost, and the need for standardized bioinformatics and data deposit guidelines, the collaborative livestock genomics community is poised to help overcome these challenges [47,109]. The future of single-cell omics technology in livestock research is promising, with many opportunities for advancing understanding of the molecular mechanisms governing complex traits to optimize breeding strategies, make advancements in animal welfare, and drive innovation in sustainable and efficient livestock production systems to meet the growing global demand for high-quality animal products.

## Figures and Tables

**Table 1:** Single-cell and single-nuclei RNA and ATAC sequencing studies published with publicly available data for cattle.

Tissue(s) or Cell Type(s)	Isolation Type	Data Type	Database and Accession *	Reference
Abomasum, ileum, liver, mammary gland, omasum, PBMC *, rectum, reticulum, rumen, salivary gland	Cell	RNA	GEO GSE176512	[6]
Adipose	Nuclei	RNA	GEO GSE211707	[7]
Embryo (8- and 16-cell stage)	Cell	RNA	GEO GSE99210	[8]
Embryo (peri-implantation)	Cell	RNA	GEO GSE234335	[9]
Embryo (SCNT)	Cell	RNA	SRA PRJNA727165	[10]
Embryo (trophectoderm)	Cell	RNA	GEO GSE200216	[11]
Fetal Gonads	Cell	RNA	GEO GSE162952	[12]
Milk Somatic Cells	Cell	RNA	ENA PRJEB73560	[13]
Muscle	Cell	RNA	GEO GSE205347	[14]
Muscle	Cell	RNA, ATAC	GSA CRA006626	[15]
PBMC *	Cell	RNA, ATAC	GEO GSE225962	[16]
PBMC *	Cell	RNA, ATAC	GEO GSE166473	[17]
Placenta (developing)	Cell	RNA	GEO GSE234524	[18]
Placenta (mature)	Nuclei	RNA	GEO GSE214407	[19]
Primary Mammary Epithelial Cells	Cell	RNA	FAANG PRJEB41576	[20]
Ruminal epithelial cells	Cell	RNA	GEO GSE166473	[21]
Satellite cells	Cell	RNA	GEO GSE184128	[22]
Satellite cells	Cell	RNA	GEO GSE211428	[23]

* Abbreviations: PBMC (peripheral blood mononuclear cells), GEO (Gene Expression Omnibus), SRA (Sequence Read Archive), ENA (European Nucleotide Archive), GSA (Genome Sequence Archive), FAANG (Functional Annotation of Animal Genomes).

**Table 2:** Single-cell and single-nuclei RNA and ATAC sequencing studies published with publicly available data for sheep.

Tissue(s) or Cell Type(s)	Isolation Type	Data Type	Database and Accession *	Reference
Conceptus	Cell	RNA	NCBI PRJNA987334	[24]
Embryo	Cell	RNA	GEO GSE185233	[25]
Ovary	Cell	RNA	GEO GSE233801	[26]
Rumen	Cell	RNA	GSA CRA007511	[27]
Rumen	Cell	RNA	NCBI PRJNA919098	[28]
Skin	Cell	RNA	GEO GSE186204	[29]
Testes	Cell	RNA	GEO GSE184343	[30]
Testes	Cell	RNA	GSA CRA005236	[31]

* Abbreviations: NCBI (National Center for Biotechnology Information), GEO (Gene Expression Omnibus), GSA (Genome Sequence Archive).

**Table 3:** Single-cell and single-nuclei RNA and ATAC sequencing studies published with publicly available data for goats.

Tissue(s) or Cell Type(s)	Isolation Type	Data Type	Database and Accession *	Reference
Fetal Gonad	Cell	RNA, ATAC	GSA CRA006304, CRA006365	[32]
Lung	Nuclei	RNA	GEO GSE183300	[33]
Ovarian Follicle	Cell	RNA	GEO GSE135688	[34]
Ovary	Cell	RNA	NCBI PRJNA1010653	[35]
Rumen	Cell	RNA	NCBI PRJNA919098	[28]
Skin	Cell	RNA	GEO GSE144351	[36]
Skin	Cell	RNA	GEO GSE182474	[37]
Skin	Cell	RNA	GEO GSE141284	[38]
Testes	Cell	RNA	GEO GSE234407	[39]

* Abbreviations: NCBI (National Center for Biotechnology Information), GEO (Gene Expression Omnibus), GSA (Genome Sequence Archive).

## Data Availability

All data discussed in this manuscript is publicly available and no new data were generated. Databases and accession numbers are listed for cattle (Table 1), sheep (Table 2), and goats (Table 3).
